# A behavioral screen for mediators of age-dependent TDP-43 neurodegeneration identifies SF2/SRSF1 among a group of potent suppressors in both neurons and glia

**DOI:** 10.1371/journal.pgen.1009882

**Published:** 2021-11-01

**Authors:** Jorge Azpurua, Enas Gad El-Karim, Marvel Tranquille, Josh Dubnau

**Affiliations:** 1 Department of Anesthesiology, Stony Brook School of Medicine, Stony Brook, New York, United States of America; 2 Department of Physiology and Biophysics, M.S. Program, Stony Brook School of Medicine, Stony Brook, New York, United States of America; 3 Department of Neurobiology and Behavior, Stony Brook University, Stony Brook, New York, United States of America; Brown University, UNITED STATES

## Abstract

Cytoplasmic aggregation of **T**ar-**D**NA/RNA binding **p**rotein **43** (TDP-43) occurs in 97 percent of amyotrophic lateral sclerosis (ALS), ~40% of frontotemporal dementia (FTD) and in many cases of Alzheimer’s disease (AD). Cytoplasmic TDP-43 inclusions are seen in both sporadic and familial forms of these disorders, including those cases that are caused by repeat expansion mutations in the *C9orf72* gene. To identify downstream mediators of TDP-43 toxicity, we expressed human TDP-43 in a subset of *Drosophila* motor neurons. Such expression causes age-dependent deficits in negative geotaxis behavior. Using this behavioral readout of locomotion, we conducted an shRNA suppressor screen and identified 32 transcripts whose knockdown was sufficient to ameliorate the neurological phenotype. The majority of these suppressors also substantially suppressed the negative effects on lifespan seen with glial TDP-43 expression. In addition to identification of a number of genes whose roles in neurodegeneration were not previously known, our screen also yielded genes involved in chromatin regulation and nuclear/import export- pathways that were previously identified in the context of cell based or neurodevelopmental suppressor screens. A notable example is *SF2*, a conserved orthologue of mammalian *SRSF1*, an RNA binding protein with roles in splicing and nuclear export. Our identification *SF2/SRSF1* as a potent suppressor of both neuronal and glial TDP-43 toxicity also provides a convergence with *C9orf72* expansion repeat mediated neurodegeneration, where this gene also acts as a downstream mediator.

## Author summary

Loss of nuclear function of TDP-43 and its mislocalization into cytoplasmic inclusions are central features to a suite of neurodegenerative disorders. We screened 2700 *Drosophila* genes to identify downstream mediators that suppress an age-dependent motor dysfunction phenotype when they are knocked down by RNA interference. We identified both previously implicated pathways and several novel genes whose knock down is sufficient to dramatically and robustly rescue TDP-43 toxicity both in neuronal and glial contexts. Notably, we demonstrate that *SF2/SRSF1*, which was previously reported as a suppressor of *C9orf72* hexanucleotide expansion repeat toxicity, also is a potent suppressor of TDP-43 mediated neurodegeneration.

## Introduction

Protein aggregation pathology of TDP-43 is a central feature in a suite of neurodegenerative disorders, including ALS, FTD and AD or those with an AD like presentation (Limbic-predominant Age-related TDP-43 Encephalopathy, LATE) [1]. In ALS in particular, TDP-43 protein pathology is seen in ~97% of patient’s post mortem tissue from affected brain regions [2]. Normally TDP-43 localizes primarily to the nucleus [3] of both neurons and glial cells, where it plays key roles in splicing, transcriptional regulation, DNA repair and transposable element suppression [4–10]. The pathological mislocalization into cytoplasmic inclusions will involve a loss of nuclear function and may produce toxic cytoplasmic effects as well. Importantly, such pathology is seen across the majority of both familial (fALS) and sporadic (sALS) cases. Familial forms of ALS that exhibit TDP-43 proteinopathy include the rare cases that are caused by mutations in the TDP-43 protein coding region [11,12], and those caused by *C9orf72* repeat expansions [13], which is the most common genetic cause of ALS and FTD in the USA.

To identify mediators of TDP-43 neurodegeneration, several previous studies have capitalized on the toxicity of overexpressing TDP-43. Such over-expression often leads to nuclear clearance and accumulation of cytoplasmic inclusions. This strategy has been successfully employed to identify genes that enhance or suppress TDP-43 aggregation and toxicity, and many of these have been validated in human disease. For instance, screening for suppressors and enhancers in yeast led to the identification of several relevant downstream mediators of TDP-43, FUS and C9ORF72 toxicity [14–17], including ATX2 [18], whose role in human disease epidemiology has since been validated [19,20]. More recently, several suppressor/enhancer screens have been performed in a *Drosophila* developmental context in photoreceptor neurons offering the advantage of being both a metazoan and neuronal context. Together, these studies have identified important core cell biological pathways including dysfunction in nucleocytoplasmic transport [17,21,22] and chromatin remodeling [23] as convergence points of TDP-43 induced pathology.

To identify downstream effectors in the context of an intact neural circuit, we conducted a behavioral screen for suppressors of age dependent decline in negative geotaxis locomotion in *Drosophila*. We used an unbiased RNAi screen of over 2700 genes and identified over 30 that suppressed the locomotion phenotype even in aged animals. This screen identified genetic pathways that have previously been implicated in neurodegeneration, as well as novel robust suppressors not previously connected to TDP-43 toxicity. Among these is the *SF2/SRSF1* gene, which is a known suppressor of C9ORF72 models of ALS/FTD [24].

## Results

### A behavioral screen to identify suppressors of TDP-43-mediated motor neuron toxicity

Expression of a codon-optimized human TDP-43 (*UAS-hTDP-43*) transgene [25] using the E49 motor neuron Gal4 (*E49-Gal4*) driver [26] is sufficient to cause [27] adult fly motor deficits and an increased rate of mortality. We noted that when both the *E49-Gal4* and the *UAS-hTDP-43* were maintained together in a single strain, the motor deficit diminished dramatically over a period of approximately five generations. This suggested that naturally occurring variants within the genetic background could be selected over time to act as modifiers of TDP-43 toxicity. We therefore postulated that individual gene knock downs might also be used effectively to identify mediators of the toxicity of TDP-43 to motor neurons.

**Fig 1:**
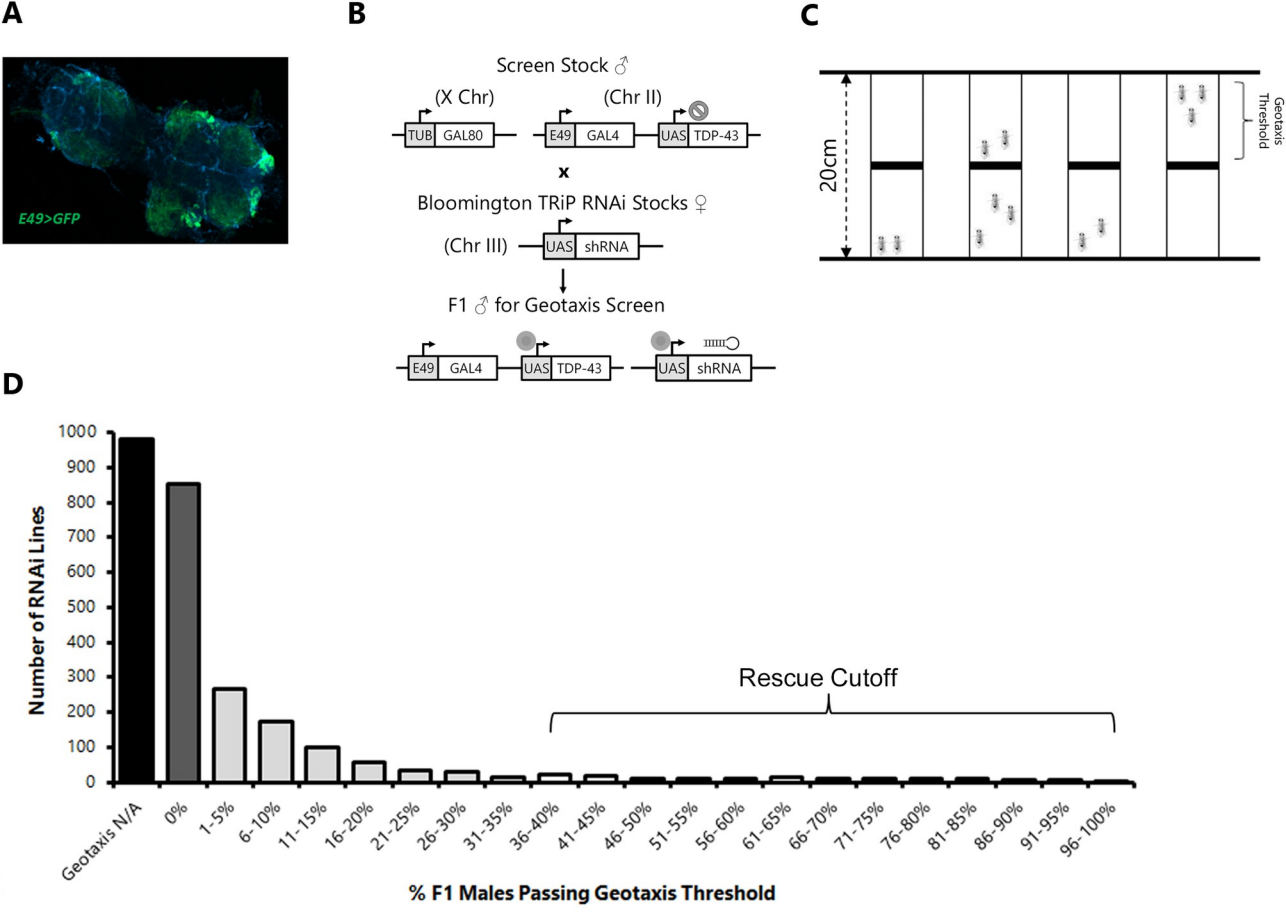
shRNA screen for suppressors of motor-neuron driven TDP-43 negative geotaxis defects. (a) Confocal microscopy image (10x objective) of dissected thoracic ganglion showing GFP expression driven by E49-Gal4. (b) Diagram of cross scheme used in screen. Males from parental strain carrying an X-linked Gal80 and autosomal E49 motor GAL4 driver (subset of motor and sensory neurons) and a codon-optimized human TDP-43 transgene on chromosome II were crossed to virgin females from candidate shRNA lines yielding F1 males lacking GAL80 and therefore expressing TDP-43 and the candidate shRNA in E49 neurons. (c) Diagram of negative geotaxis setup for behavioral rescue assay. (d) Histogram showing shRNA lines (y-axis) binned by geotaxis performance (% of flies crossing 10cm threshold in 10 seconds, x-axis) across all tested shRNA lines. Black bar indicates crosses for which geotaxis was not possible due to low number of eclosing males. Dark gray bar indicates lines for which sufficient flies were obtained, but none of them were capable of climbing to the threshold within 10 seconds. Light gray bars are histogram bins in which at least 1 fly was able to cross threshold within 10 seconds. The horizontal bracket indicates the cutoff for retesting as a candidate suppressor.

To avoid the confounding impact of selected suppressors emerging among natural variants in the strain, we created a new recombinant stock that contained not only the *E49-Gal4* driver (expression pattern is shown for GFP in **Fig 1A**) and the *UAS-TDP-43* transgene insertion, but also a ubiquitously expressed *tub-Gal80* repressor (Gal80) on the X chromosome. The presence of the Gal80 repressor in this strain prevents expression of the *UAS-TDP-43*, and thereby prevented accumulation of modifying alleles within the strain. However, when males of this strain are crossed to wild-type females, the *tub-Gal80* is easily segregated, in male progeny, from the *E49-Gal4* and *UAS-TDP-43* transgenes which reside on autosomes. We found that such male offspring consistently and stably produced severe adult motor defects even after the parental stock was maintained for many (>20) generations. We used this *Gal80;E49>TDP-43* strain to conduct an unbiased genetic screen for suppressors of the adult locomotion defect. By crossing males from this parental strain with females that contained individual UAS-driven shRNAs typically targeting a single gene, we screened the impact of each shRNA on locomotion in approximately one week old male progeny that had lost the Gal80 repressor (**Fig 1B**).

The design of this screen involves expressing both the *UAS-TDP-43* and the *UAS-shRNAs* via the same *E49-Gal4* driver. We were cognizant of the fact that the presence of two loci that contain the same Gal4 responsive UAS enhancer might titrate the available Gal4 transcription factor available for each. To mitigate against the possibility that differences in such titration across shRNAs could yield false positive or negative effects, we limited this screen to shRNAs from a single collection that all use an identical “VALIUM20” vector as their RNAi expression system. Additionally, we only used the subset of strains [28] that contained a VALIUM20 *UAS-shRNA* inserted into a specific AttP2 landing site on the third chromosome. This collection of VALIUM20 shRNA insertions at this defined chromosomal location consisted of 2724 strains, generally targeting a specific *Drosophila* genomic locus. We crossed females from each of these 2724 *UAS-shRNA* containing strains with males from our tub-*GAL80;E49>TDP-43* strain (**Fig 1B**). We then quantified negative geotaxis locomotion behavior [29] among F1 male offspring from each cross at approximately 1 week of adult age. In order to scale up the behavioral screening, we designed a simple apparatus (**Fig 1C**) that allowed multiple strains to be tested in parallel. We established a stringent locomotion value (35% of animals crossing 10cm marker within 10seconds) to identify candidate suppressors, and then each candidate suppressor was re-screened with an independent cross to validate the findings from the primary screen (**Fig 1**). This also permitted us to calculate both the overall false positive and false negative rates (**S1 Table**, false positive rate ~2%, false negative rate negligible). The vast majority of the crosses yielded F1 progeny that had severe locomotion defects. In many cases there either were too few surviving male progeny to screen, or we found that none of the flies were able to climb past the 10cm marker within the allotted time (**Fig 1D; S2 Table**). We identified 33 shRNA crosses (1.24% of total) that resulted in greatly improved locomotion, with greater than 35% of the animals crossing threshold in both the primary screen and the independent validation crosses (**Table 1**; **S2 Table**). As a further validation, 22 of these screen hits were retested after being equilibrated (**S1 Fig**) with 5 generations of back-crossing into the w^1118^(isoCJ1) genetic background (a derivative of Canton S) both to match that of the *GAL80;E49>TDP-43* strain and to test for robustness of the effects in a new genetic background. The suppression of the negative geotaxis behavioral defect persisted with 21/22 tested in this second genetic background (**Table 1**).

**Table 1:** List of genes targeted by TDP-43 toxicity rescuing shRNA lines.

Flybase Symbol	Name	Putative Mammalian Orthologues	Gene Ontology/Function	Bloomington TRiP Stocks	Replicated After Introgression
*Chd1*	*Chromodomain-helicase-DNA-binding protein 1*	*CHD1*, *CHD2*	Chromatin remodelling	34665	Y
*e(y)3*	*enhancer of yellow 3*	*PHF10*	Chromatin remodelling	32346	N/A
*polybromo*	*polybromo*	*BAF180*	Chromatin remodelling	32840	Y
*ash1*	*absent*, *small*, *or homeotic discs 1*	*ASH1L*	Chromatin remodelling	33705	Y
*enok*	*enoki mushroom*	*KAT6A*	Chromatin remodelling	40917, 41664	Y (both)
*br*	*broad*	*-*	Chromatin remodelling	33641	N/A
*Br140*	*Bromodomain-containing protein*, *140kD*	*BRPF1*	Chromatin remodelling	56034	N/A
*mor*	*moira*	*BAF170 / SMARCC2*	Chromatin remodelling	34919	N/A
*msk*	*moleskin*	*IMP7*	Nuclear import/export	33626	Y
*nito*	*spenito*	*RBM15*	Nuclear import/export	34848	Y
*sbr*	*small bristles*	*NXF1*	Nuclear import/export	34945	**N**
*Hel25E*	*Helicase at 25E*	*UAP56*	Nuclear import/export	33666	Y
*Su(Tpl)*	*Suppressor of Triplolethal*	*ELL2*	Elongator	33399	Y
*SF2*	*Splicing factor 2*	*SRSF1*	Splicing	32367	Y
*Hsp70B (Ba/Bb/Bbb/Bc)*	*Heat-shock-protein-70B (Ba/Bb/Bbb/Bc)*	*HSPA1A*	Heatshock	33948	Y
*Gγ30A*	*G protein subunit γ at 30A*	*GNG13*	Signal transduction / G protein	34484	N/A
*MED8*	*Mediator complex subunit 8*	*MED8*	Mediator complex	34926	Y
*MED11*	*Mediator complex subunit 11*	*MED11*	Mediator complex	34083	Y
*MED15*	*Mediator complex subunit 15*	*MED15*	Mediator complex	32517	Y
*MED21*	*Mediator complex subunit 21*	*MED21*	Mediator complex	34731	N/A
*MED22*	*Mediator complex subunit 22*	*MED22*	Mediator complex	34573	N/A
*MED27*	*Mediator complex subunit 27*	*MED27*	Mediator complex	34576	N/A
*MED28*	*Mediator complex subunit 28*	*MED28*	Mediator complex	32459	Y
*MED31*	*Mediator complex subunit 31*	*MED31*	Mediator complex	34574	Y
*CG7309*	*-*	*SCL13A1*	Transmembrane transport	44653	N/A
*CG8034*	*-*	*SCL16A1*	Transmembrane transport	32340	N/A
*CG2186*	*-*	*RMB33*	RNA-binding	33750	Y
*CG30379*	*-*	*GRINA / Lifeguard1*	Ca2+ homeostasis	36679	Y
*CG9451*	*-*	*ACP2*, *ACP3*, *ACP4*	Acid phosphatase	34545	Y
*e(y)1*	*enhancer of yellow 1*	*TAF9*	Transcription Factor	32345	Y
*TAF1*	*TBP-associated factor 1*	*TAF1*	Transcription Factor	32421	N/A
*Tomb*	*tombola*	*-*	Male meiosis	36669	Y

Gene ontology was surmised from FlyBase and PubMed overviews of the mammalian orthologues. Function/ontology for unnamed genes should be considered speculative. The last column indicates the Bloomington Drosophila Stock Center number for the shRNA line with an insert targeting the gene. The shRNA for Hsp70B targets 4 genomic loci, as the Hsp70B gene has four functional duplications (Ba, Bb, Bbb, and Bc).

These TDP-43 suppressors fall into several broad functional categories. First, we identified a number of novel genes that have not previously been implicated in neurodegeneration and a relatively large group that are components of the Mediator complex. Mediator proteins integrate signals between transcription factors (TFs) and RNA polymerase, with different subunits showing some specificity for interactions with distinct subsets of TFs [30]. Second are those involved in cell biological functions that are well established to play a role in neurodegeneration. These include chromatin remodeling factors [23,31,32] and those involved in nucleocytoplasmic transport [17,23,33–36]. In the latter category, the msk/importin-7 gene is of interest because the sign of its effect is opposite from what one would predict if it were involved in transport of TDP-43 (see discussion). Finally, we highlight that the SF2/SRSF1 gene was identified among our TDP-43 suppressors. This is notable because this gene has been found as a downstream effector in the context of C9orf72 repeat expansion toxicity [24].

### Motor neuron-specific CRISPR/Cas9 knockout of msk/importin-7 and SF2/SRSF1 suppresses negative geotaxis behavior

**Fig 2:**
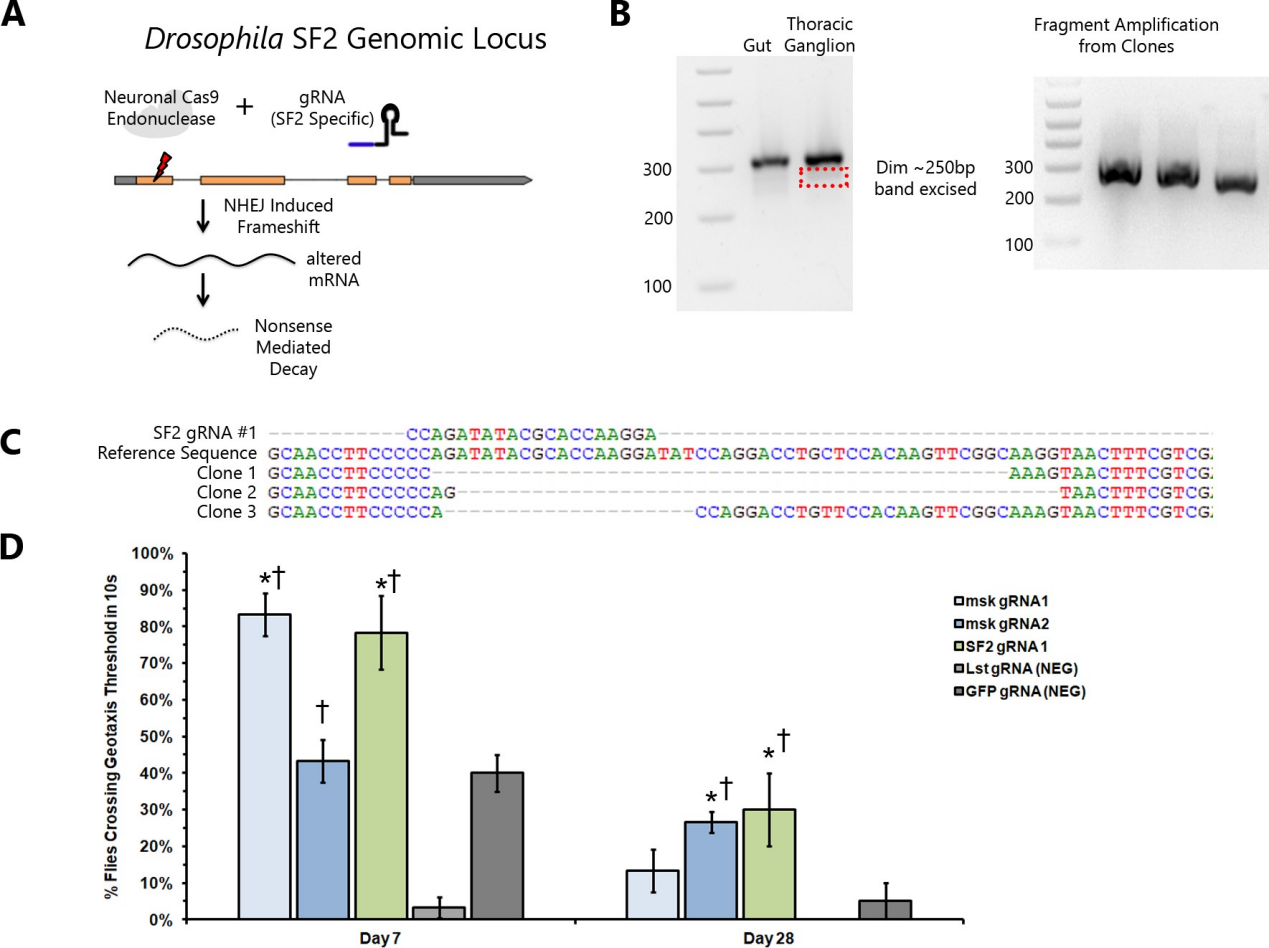
Motor neuron specific CRISPR/CAS9 knock-out of msk/importin-7 and of SF2/SRSF1 are each sufficient to prevent TDP-43 mediated defects in negative geotaxis. (a) Overview of CRISPR/Cas9 knock out strategy. gRNAs specific to *SF2/SRSF1* or *msk/importin-7* (see **S3 Table**) are expressed constitutively in all cells by a U6 promoter plasmid integrated into the AttP2 landing site. Cas9 expression is restricted to E49 neurons, limiting editing to those cells. (b) Gel electrophoresis of PCR products from dissected tissues of male F1 offspring. Gel 1 Lanes: DNA ladder, Gut amplification product (no editing), thoracic ganglion amplification product (editing in some neurons). A faint band corresponding to reduced size predicted from edited products was excised and cloned into a sequencing vector. Gel 2 Lanes: DNA Ladder, and three independent PCR amplifications from sub-cloned edited inserts. These show variable size corresponding to stochastic NHEJ editing of target gene. (c) DNA sequence of edited fragments. Sequences from top to bottom: guide RNA targeting SF2 gene, reference SF2 sequence, clones 1–3 showing variable deletions caused by NHEJ repair. (d) Negative geotaxis of F1 *E49>TDP-43*, *Cas9; U6-gRNA* males over an aging time-course. Y axis shows % flies (average of three trials) crossing a 10cm threshold after 10 second (negative geotaxis testing). X axis indicates time point (7 days or 28 days post eclosion). Bar color indicates gene targeted by gRNA. * P < 0.001 by Student’s T test in comparisons to GFP control. † P < 0.001 by Student’s T test in comparisons to *lst* control.

We used CRISPR/Cas9 gene editing as an independent means to validate that TDP-43 toxicity to motor neurons can be suppressed by knockout of *SF2*/*SRSF1* and *msk*/*importin-7*. (**Fig 2A**) We designed guide RNAs (gRNAs) targeting these genes and we generated transgenic animals that constitutively express these gRNAs by integrating into the same AttP2 landing site that was used for the shRNA screen. As controls, we used gRNAs targeting either GFP or the *lst* gene, which was not identified as a suppressor in our shRNA screen and is not known to be expressed in motor neurons. We tested the impact of expressing each of these gRNAs in animals that also contained the *E49-Gal4* line, *UAS-TDP-43* and *UAS-Cas9* transgenes.

As a first test of the efficacy and specificity of our cell type specific gRNA targeting strategy, we dissected thoracic ganglia, which contain motor neurons vs gut as a control. DNA from these tissues was extracted and the region flanking the gRNA target sites were queried by PCR and sequencing. Despite the presence of unedited non-motor neuron cells in the thoracic ganglion sample, we were able to detect and sequence internal deletions in the region that is targeted by the *SF2*/*SRSF1* gRNA. In contrast, such deletions were not detected from gut (**Fig 2B and 2C**).

We next tested the effects on negative geotaxis behavior of the gRNAs against GFP, *lst*, *mks*/*importin-7* and *SF2*/*SRSF1*. We quantified negative geotaxis behavior over the course of a 4 week aging time period and found that relative to the gRNAs targeting GFP or *lst*, the gRNA that targets the *SF2*/*SRSF1* gene suppressed the effects of TDP-43 in animals that were 7, or 28 days old (**Fig 2D**). Similarly, with *msk*/*importin*-7, each of two independent gRNAs were sufficient to suppress the negative geotaxis defect caused by TDP-43 expression in motor neurons, with *msk* gRNA1 showing significant effects at 7 and 28 days and gRNA2 showing significant effects at 28 days of age (**Fig 2D**).

### Suppressors prevent age-dependent decline of negative geotaxis behavior

Because neurodegenerative phenotypes exhibit a marked age-dependence, we tested whether our identified suppressors were capable of ameliorating the effects of motor neuron TDP-43 toxicity over an aging time course. For this experiment, we tested 20 of the suppressors that had been equilibrated into a common genetic background (**S1 Fig**; **Table 1**). These were selected to span each of the categories of gene function among the hits from the primary screen. In parallel, we tested a series of 6 negative controls shRNAs. As negative controls, we used UAS-shRNAs against three genes (*ecd*, *SirT7* and *CG8005*) that had not impacted negative geotaxis in our primary screen. And we also tested 3 different shRNA lines that targeted heterologous transcripts that are not present as endogenous sequences in the fly genome (mCherry, GFP, and Luciferase). Together, these 6 shRNAs controlled for several potential confounds, including the possibility that providing a second UAS-driven transgene might provide functionally relevant reduction in the levels of the *UAS-TDP-43* transgene. We view this possibility as unlikely because with several of our shRNA suppressors, we observe robust expression of GFP in animals that contain the *E49-Gal4* driver, *UAS-GFP* and a *UAS-shRNA* compared with controls that do not contain the shRNA (**S2 Fig**). In addition to providing a robust set of controls for the possibility of titrating the expression of *UAS-TDP-43*, these 6 shRNAs also controlled for the possibility that overwhelming the shRNA silencing system might impact TDP-43 neurotoxicity. As with the 20 shRNA suppressors, the 6 control shRNAs are inserted at the common AttP2 PhiC31 landing site, and were equilibrated into the same genetic background.

**Fig 3:**
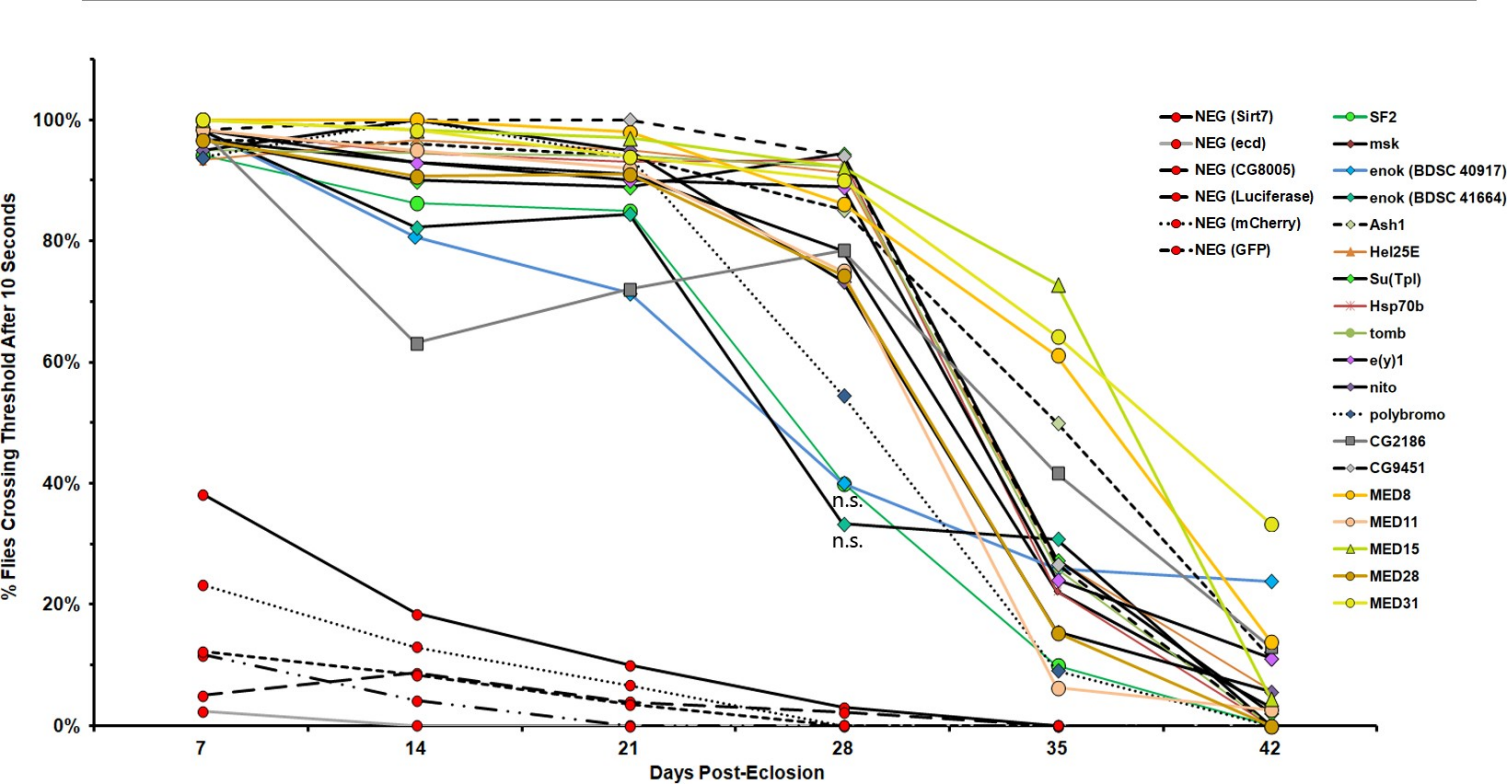
shRNA suppressors rescue the negative geotaxis defect caused by motorneuron-TDP-43 and prevent further age-dependant decline. 25 shRNAs were introgressed into a common wild type *Drosophila* genetic background w^1118^ (iso CJ1), a derivative of CS, and crossed to the TDP-43 screen flies described in **Fig 1A**. Negative geotaxis was assayed among F1 progeny of each cross beginning 7 days after eclosion and each week thereafter. Negative control shRNAs (Red circles) exhibited severe climbing defects that further deteriorated with each subsequent week. Suppressor shRNAs (non-red data points) ameliorated the effects of TDP-43 in young animals (7 day old) and prevented decline in climbing behavior until the 28 day time point or later. Statistical significance was determined by paired Student’s T-test between suppressor shRNAs and the mCherry negative control shRNA, adjusted for multiple testing by Bonferroni correction. The P value threshold for significance was P < 0.0025. Due to data density on the graph we have indicated significance directly except as noted below. For 7 days after eclosion, all shRNAs were significantly different (P < 0.00001) except for *sbr* (P = 0.46). Statistical significance was also tested 28 days after eclosion, all shRNAs were significantly different (P < 0.01) except *sbr*, *enok* ^40917^ (blue diamond/blue line data point, P = 0.016) and *enok* ^41664^ (green diamond/black line data point, P = 0.0028). Non-significance of the *enok* data points are indicated by an “n.s.” on the graph.

Negative geotaxis behavior was quantified among F1 males for each of these 26 crosses beginning 1 week after eclosion, and every 7 days thereafter (**Fig 3**). Each of the six negative control shRNAs exhibited severe defects in locomotion at 1 week post eclosion, consistent with their failure to suppress the geotaxis assay in the primary screen. Animals carrying each of these control shRNA also exhibited rapid deterioration in climbing ability at subsequent age time points. By the end of 6 weeks, most of the surviving flies in the negative control groups were largely incapable of climbing (**Fig 3**, red data points). In contrast, 19 out of the 20 shRNA lines that we identified as suppressors in the primary screen exhibited robust climbing ability, with nearly 100% of animals crossing the 10cm distance within 10 seconds. And remarkably, this amelioration of the climbing defects caused by TDP-43 expression in motor neurons was largely resistant to age over a 6 week period (**Fig 3**). The only exception to this is the shRNA targeting the *sbr/NXF1* gene, which was identified as a suppressor in the primary screen (**Table 1**; **S2 Table**) but whose suppression was eliminated when it was crossed into the w^1118^ (iso CJ1) background.

### SF2/SRSF1 and the majority of shRNAs tested strongly suppress TDP-43 toxicity in a glial context

The primary shRNA screen described above was conducted with TDP-43 expression in motor neurons. But there is a growing recognition of the importance of glial cells in neurodegeneration [37], and we and others have documented that glia play a major role in fly models that involve TDP-43 [38–41]. We therefore wondered whether any of the TDP-43 suppressors that we had identified in neurons also would ameliorate the toxicity of TDP-43 when expressed in glial cells. To test this, we selected a panel of the shRNA suppressors targeting 10 genes that span the functional categories identified. To test the effects of knocking down each of these 10 genes in flies that express a non-codon optimized TDP-43 transgene in glia (see **S5 Table**), we made use of an inducible glial expression system in which a temperature sensitive Gal80 repressor is used to modulate the expression of TDP-43 and the shRNA, under control of the Repo-Gal4 that expresses in most glial cells. We have previously shown that inducing pan glial TDP-43 expression after development is sufficient to greatly reduce lifespan, and cause both glial death and non-cell autonomous toxicity of TDP-43 expressing glia that also kills adjacent neurons[41]. We crossed these tub-*GAL80TS*, *Repo-Gal4; UAS-TDP-43* animals (hereafter referred to as *Repo*^*TS*^*>TDP43*) with each of the *UAS-shRNAs* and examined lifespans of the F1 offspring after heat-shift induction of TDP-43.

**Fig 4:**
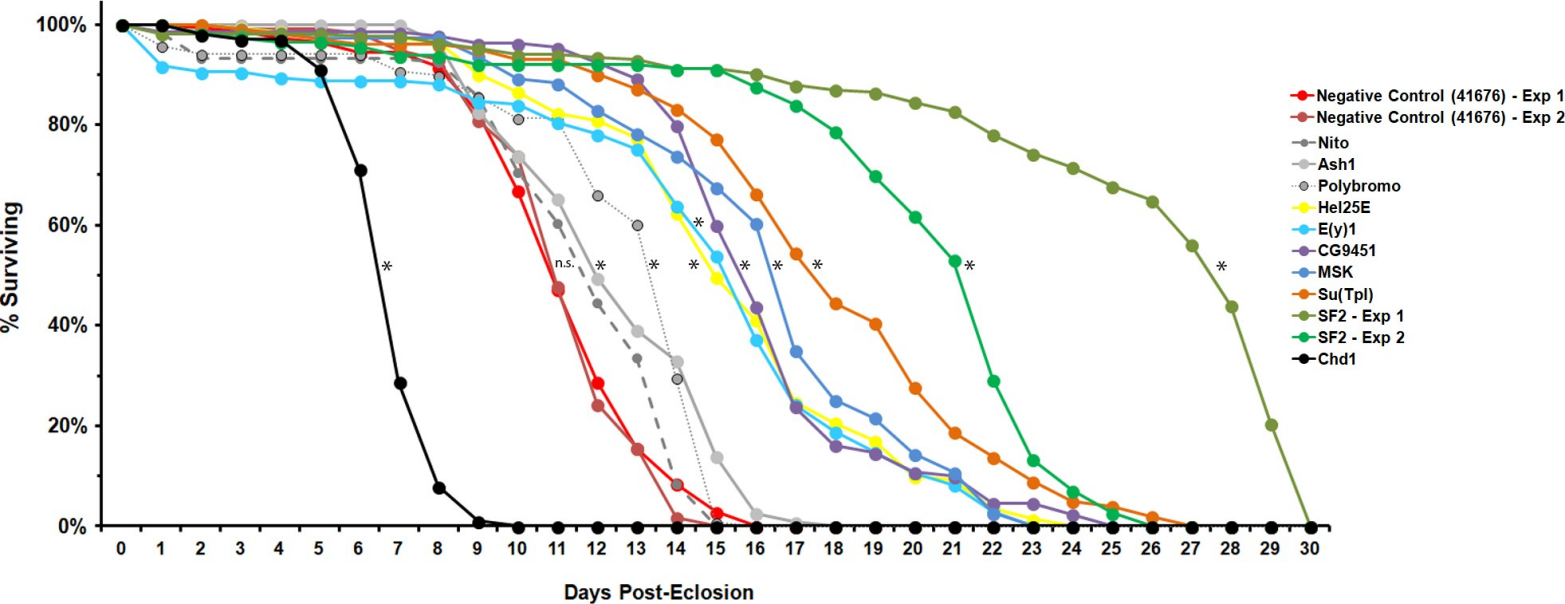
Multiple suppressors, including *SF2/SRSF1*, potently extend lifespan of flies expressing TDP43 in glia. 11 shRNAs that had been equilibrated into a common genetic background w^1118^ (iso CJ1) were crossed to the *RepoTS>TDP43* strain that expresses a UAS-TDP-43 in glia under control of a temperature sensitive Gal80 and the Repo-Gal4 line. These animals induce human TDP-43 in glia after being shifted to 30°C, the non-permissive temperature. F1 males and virgin females were placed into separate vials for aging (20 animals per vial). Time since induction (shift to 30°C) is shown in the X-axis. % survival is shown on the Y-axis. Red data points show 2 independent replicates with a negative control shRNA (shRNA to *ecd*, introgressed from BDSC 41676) which exhibit nearly complete mortality by 2 weeks. The *Chd1* shRNA (black data points) exacerbates mortality in this glial context, which is in contrast to its effect in motor neurons. *Nito* (gray large dash line) did not extend lifespan significantly after adjusting for multiple testing, nor was the potential effect large. The *ash1* and *polybromo* shRNAs (gray solid and small dashed lines) yield modest but significant increases in lifespan. Each of the other shRNAs (colored data points) increased lifespan dramatically including two independent experiments where the *SF2* shRNA extended lifespan by 2.5–3 fold. Statistical significance (indicated by * on graph) was determined by Kaplan-Meier estimator pairwise comparisons to the negative controls, using Bonferroni correction for multiple testing (k = 11, P < 0.004 for significance). P values were <0.00001 for all comparisons except *nito* (P = 0.016, indicated by “n.s.” on the graph).

Control flies expressing an shRNA against the *ecd* gene (shRNA 41676), which did not impact the motor neuron phenotypes with *E49>TDP-43* also had no impact on the severely shortened lifespan of *Repo*^*TS*^*>TDP-43*. Two independent experiments in which this control shRNA were expressed exhibited median survival of ~11 days (**Fig 4** red lines). In contrast, we observe significant lifespan extension with 9/10 shRNAs that target genes identified in the motor neuron suppressor screen. The effect was modest with 2/10 shRNA suppressors (*polybromo*, *ash1* –**Fig 4** gray lines, not significant for *nito*) and was more robust for 6/10 (**Fig 4**). It also is worth noting that one shRNA, against the *Chd1* gene, was identified as a suppressor in the context of motor-neuron toxicity (**Table 1**) but has the opposite sign in the context of glial TDP-43 toxicity. The shRNA against *Chd1* significantly decreased lifespan of the *Repo*^*TS*^*>TDP-43* animals. Thus, action of this gene on TDP-43 toxicity may be cell type dependent. Finally, the most potent suppressor in this glial context was *SF2*/*SRSF1*, which yielded a median survival of ~22 and ~29 days in independently replicated experiments (**Fig 4** green lines). Despite the expression of TDP-43 in most glia, the lifespan of animals that express *SF2*/*SRSF1* shRNAs is near that expected from wild type animals under these temperature conditions (30°C). Because the suppression that we observed was so robust with SF2/SRSF1 knock down, we used Western immunoblotting to verify that TDP-43 protein was equivalently expressed in these animals. We found that 5 days after induction, the levels of TDP-43 in the presence of the *SF2/SRSF1* shRNA are indistinguishable from those seen with the mCherry control shRNA (**S3 Fig**). This supports the conclusion that SF2/SRSF1 mediates toxicity of TDP-43 rather than its production.

## Discussion

Overexpression of either wild type or disease alleles of TDP-43 triggers concentration dependent TDP-43 protein aggregation and can lead to TDP-43 clearance from the nucleus and appearance of hyperphosphorylated cytoplasmic inclusions. This approach has been used in yeast [13,14], mammalian cell culture [42–44], and animal models including nematodes [45], flies [25,38,40,41,46,47], and rodents [48–52], and has led to identification of downstream functional defects that have been validated in human tissues. These include defects in splicing [4,6,7,53], nuclear-cytoplasmic localization [17,22,33], DNA damage signaling [40,41,54,55] and expression of retrotransposons and endogenous retroviruses [10,40,41,56–60]. Many of the above findings are derived from high-throughput screens to identify genetic modifiers of TDP-43 toxicity. But to date, no unbiased screen has been conducted in the context of an age-dependent neurological phenotype that emerges from a functioning neural circuit. Moreover, no such screen has been attempted in the context of glia, which play a fundamental role in disease progression [37].

We used over-expression of human TDP-43 in a subset of *Drosophila* motor neurons as the basis for an shRNA knockdown screen to identify suppressors of TDP-43 neurotoxicity. Young (newly eclosed) adult flies with such motor neuron expression of TDP-43 exhibit mild defects in climbing behavior in a negative geotaxis assay, but this defect becomes dramatically worse with age. Using this approach, we screened a collection of shRNAs that covered approximately 15–20% of protein coding genes in the *Drosophila* genome, and found that approximately 1% of shRNAs can abrogate TDP-43 toxicity. For the majority of these, shRNA knockdown also potently prevented the age-dependent decline in locomotion. Additionally, for most of the cases tested, effects on lifespan from TDP-43 expression in glia also was ameliorated.

The majority of suppressors described here have not previously been implicated in neurodegeneration. But several of the hits have been identified in previous screens for TDP-43 suppressors, and a number of others play roles in cellular pathways that have established impact on TDP-43 related neurodegeneration. For example, we identified 8 genes with roles in chromatin remodeling. These include several specific genes that have been found in previous screens. *Chd1* and *moira*, for instance, are chromatin remodeling factors that were identified [23] in a previous screen in flies for modifiers of a TDP-43 induced rough eye phenotype. It is worth noting, however, that in this previous study, downregulation of *Chd1* exacerbated the toxicity of TDP-43 to photoreceptor neurons, leading to a more severe rough eye phenotype. This is the opposite sign of effect that we observed in the context of our motor-neuron based behavioral screen. On the other hand, when we screened hits in the context of effects of glial TDP-43 on lifespan, we found that *Chd1* knockdown exacerbated the toxicity, consistent with the sign of effect seen in the previous study. This drives home the importance of cellular context, and the importance of testing impact within both glia and neurons.

In the case of the 8 chromatin modulators identified as suppressors, their functional roles may also provide some mechanistic insight. With the exception of *Chd1*, which promotes chromatin silencing, the 7 other genes in this category all promote open chromatin. *E(y)3*, *polybromo*, *ash1*, *moira*, *br* and *br140* are all part of the *trithorax* and SWI/SNF (Brahma) complexes and *enok* is a histone acetyltransferase that interacts with *trithorax* group genes and also promotes open chromatin. With each of these, TDP-43 toxicity is ameliorated by shRNA knock down, suggesting that chromatin silencing normally helps keep TDP-43 toxicity in check. This idea is consistent with the hypothesis that loss of silencing of retrotransposons and endogenous retroviruses contributes to TDP-43 toxicity [10,40,41,56,57,60].

A second category of suppressor identified here are factors involved in basal transcription. This includes 8 components of the Mediator complex, and several basal transcription factors. Although it is difficult to distinguish specific vs artifactual causes for identification of factors regulating fundamental aspects of transcription, it is worth pointing out that we also identified the transcription elongation factor, *Su(Tpl*), which was found in a previous rough eye suppressor screen [61]. In that study, the authors present compelling evidence that in the presence of TDP-43 pathology, elongator complexes aberrantly express small nucleolar RNAs. Our identification of *Su(Tpl)* suggests that a similar process could be at play in motor neurons and glia.

The third major category of suppressor includes genes with known roles in nuclear import/export control, a pathway that was also convergently identified in several previous screens [17,22], supporting the idea that defects in nuclear-cytoplasmic shuttling may be responsible for cytoplasmic mis-localization of TDP-43. We were intrigued that *msk*, one of the nuclear import factors that we identified is the *Drosophila* orthologue of Importin 7, a nuclear import protein that has a role in nuclear entry of HIV retrovirus [62,63] and LINE1 retrotransposons [64]. Thus, as with the chromatin silencers discussed above, this provides a possible link to involvement of retrotransposons and endogenous retroviruses.

Finally, we identified *SF2/SRSF1* as a particularly robust suppressor. We found that knock down of *SF2*/*SRSF1* abrogated the locomotion defect caused by TDP-43 expression in motor neurons, prevented the age-dependent decline in climbing ability out to 6-weeks of age, and potently extended lifespan in animals that over-express TDP-43 in glial cells. In fact, glial knock down of *SF2*/*SRSF1* is sufficient to all but eliminate the severe lifespan effect seen in animals that overexpress TDP-43 in glia. This RNA binding protein functions in both splicing and as a nuclear export of adaptor for RNA export. It has previously been identified in a *Drosophila* screen for suppressors of a rough eye phenotype caused by *C9orf72* hexanucleotide expansion repeat RNA [24]. In that study, the authors determined that *SF2*/*SRSF1* knock down could suppress the toxicity of a *C9orf72* repeat RNA that encoded dipeptide repeats. This was demonstrated both in the *Drosophila* context and in a co-culture system in which astrocytes from *C9orf72* derived patient fibroblasts are grown with motor neurons. In the induced astrocyte context, knock down of *SF2*/*SRSF1* was sufficient to ameliorate the toxicity of patient derived astrocytes to motor neurons. This is reminiscent of our observation of highly potent suppression of the effect on lifespan of glial TDP-43 expression in flies, an effect that we previously have documented to involve toxicity of the glia to neurons [41]. In the case of *C9orf72*, the effect of *SF2/SRSF1* knock down was shown to be mediated by modulation of the export of the repeat RNA to the cytoplasm. The reduced repeat RNA export in turn was proposed to reduce the repeat associated translation to produce toxic dipeptide repeats. In fact, *SF2*/*SRSF1* knockdown was not sufficient to significantly ameliorate the toxicity of arginine rich dipeptides expressed from non-repeat RNAs[24], consistent with the interpretation that the relevant impact of *SF2*/*SRSF1* is to aid export of the repeat RNA to the cytoplasm.

Our identification of *SF2*/*SRSF1* as a suppressor of TDP-43 toxicity in neurons and glial cells in *Drosophila* suggests convergence with the effects previously observed on the *C9orf72* repeat. In principle, this convergence could reflect the fact that *C9orf72* patients typically exhibit TDP-43 cytoplasmic inclusions [13]. Indeed in patient brain tissues, TDP-43 protein pathology often co-localizes with that of the GR dipeptide repeat [65]. Furthermore, animal models of *C9orf72* expansion toxicity also can exhibit TDP-43 proteinopathy [66]. On the other hand, we find that knock down of *SF2*/*SRSF1* has strong suppressive effects on the toxicity of TDP-43 that occurs in the absence of any *C9orf72* repeat RNAs. So in this case, effects of *SF2/SRSF1* knock down on toxicity of TDP-43 is independent of any impact on *C9orf72* repeat RNA export. The mechanism by which *SF2/SRSF1* knock down reduces toxicity is unknown. Previous work in *Drosophila* [67] reported that knockdown of *SF2/SRSF1* was sufficient reduce expression from a hTDP-43 transgene when it contained its own 3’UTR that includes an element called *TDPBR (TDP-43 Binding Region)*, which is believed to be responsible for its auto-regulation. Based on these findings, they proposed that SF2/SRSF1 may modulate TDP-43 autoregulation. But this is not likely to be an explanation for the potent suppression that we observe with SF2/SRSF1 knockdown because the TDP-43 construct that we utilized here does not contain the auto-regulatory region, but instead contains a heterologous 3’ UTR from alpha-tubulin [68]. Moreover, the levels of TDP-43 protein that we detect in the presence of the SF2/SRSF1 shRNA are indistinguishable from controls (**S3 Fig**). *SF2/SRF1* is a multifunctional protein, involved in splicing regulation, RNA export, and other aspects of RNA metabolism- for example, there is evidence that suggests that *SF2/SRSF1* can also bind the TAR region of HIV [69], which is the original function identified for TDP-43. Given the non-cell autonomous effects of TDP-43 toxicity, we also cannot rule out the possibility that the suppression observed with knock-down of *SF2/SRSF1* is mediated by reducing deleterious effect of TDP-43 toxicity to neighboring glial or neuronal cells [41].

We have used a genetic screening approach to identify suppressors of TDP-43 toxicity in *Drosophila*. Unlike previous screens, we used an age-dependent behavioral defect as a primary readout. This age-dependent effect on a neurobehavioral readout may enrich for effects that are related to the age-dependent degenerative rather than neurodevelopmental effects, but also is sensitive to disruptions to the interactions among each of the relevant cell types to a locomotion circuit, including motor neurons and glia. One open question not addressed in this study is whether the toxicity of TDP-43 expression in motor neurons is accompanied by neuronal loss, and whether the mechanisms of toxicity are the same in glia and neurons. Previous work in *Drosophila* has not established loss of motor neurons, but there is evidence of deleterious synaptic remodeling at the neuromuscular junction upon ectopic hTDP-43 expression [70]. Nonetheless, our secondary screen for suppression of toxicity to glia also reveals that many of the same genetic pathways participate in toxicity to glia and neurons. In the context of glial TDP-43 toxicity, we have previously reported that both glial cells and neurons are lost [40,41]. Our screen reveals convergence with several key findings from previous screens, namely involvement of chromatin silencing and nuclear/cytoplasmic shuttling, but also identifies genes not previously associated with neurodegeneration. Finally, the identification of *SF2/SRSF1* provides a point of convergence between TDP-43 proteinopathy and *C9orf72* familial ALS/FTD and points to unexplored mechanistic underpinnings of that overlap.

## Materials and methods

### Negative geotaxis of shRNA and CRISPR/Cas9 screening systems

Male *GAL80;E49>TDP-43* animals were crossed to females that contained either the UAS-shRNA or the gRNA and UAS-Cas9. In each case, Male F1 animals that had lost the Gal80 (both contained the E49-Gal4, the UAS-TDP-43 and either the UAS-shRNA or the gRNA and the UAS-Cas9) were collected shortly after eclosing and transferred to fresh vials with food. Behavioral testing took place between 12PM and 4PM in an isolated environmentally controlled room. Lighting conditions were kept as consistent as possible for all geotaxis screening. For the assay, a maximum of 20 flies were quickly transferred (without being anesthetized) by tapping the housing vial and transferring to an assay vial without food. Flies were placed into two 95mm vials whose open mouths were held together by black plastic gaskets. A frame capable of holding ten sets of vial pairs was used to simultaneously tap flies to the bottom of the vials. A timer and video camera were started prior to tapping the flies down. Video of climbing activity was recorded for three trials per experiment. Scoring of geotaxis was performed by determining the percentage of the flies in each vial that crossed to the top vial (10cm) within 10 seconds (recording proceeded for 15 to 20 seconds).

### Lifespan in glial expression system

Male and virgin female flies were collected over a period of 72 hours. Groups of approximately 20 flies were placed in each of 3 separate vials (n = 60 for most assayed genotypes). Males and females were tested separately. Flies were kept in temperature and humidity controlled incubators. To induce TDP-43 expression in glia, the flies were transferred to a 30°C incubator. Flies were flipped to vials of new food every other day to avoid mortality from becoming stuck in food and dead flies were removed and counted every day.

### CRISPR genome editing

Candidate gRNAs were designed by a publicly available computational prediction method https://www.flyrnai.org/crispr3/web/ [71]. For each gene of interest, 5 candidate sequences were chosen from the program output, and further filtered. A positive strand target and a negative strand target were chosen. Sequences with internal U6 terminators were excluded. All sequences listed include PAM sites (3 terminal base pairs) and were excluded from ordered oligos. Sequences were chosen on the basis of high likelihood of cutting (Housden and Machine scores) and having a high probability of inducing a frameshift after NHEJ repair. Sequences used can be found in **S3 Table**.

Oligos (**S4 Table**) were ordered from IDTDNA and included overhangs for Bbs1 restriction sites. Oligos were annealed and cloned into a linearized pCFD3.1 vector [72], cloned and purified using Qiagen EndoFree plasmid Maxiprep kit, sequenced for confirmation and sent for microinjection to BestGene Drosophila Injection Services. Transgenic U6-gRNA flies were introgressed and homogenized (pCFD3.1 was integrated into AttP2 site on Chromosome 3) by screening for the *white* phenotype rescue. CRISPR cutting in motor neurons was assessed as described in **Fig 4B**. Screening primers can be found in **S3 Table**.

### Cas9 deletion confirmation PCR, fragment cloning and sequencing

Cas9 gRNA F1 Male flies were anesthetized and pinned to a dissecting plate. The gut and thoracic ganglia were separately dissected from 10 flies and placed into DNA extraction buffer, homogenized, and treated with Proteinase K. PCR amplification using KAPA2G Fast HotStart Genotyping Mix (Kapa Biosystems) with flanking oligos (**S4 Table**) was performed for 30 cycles then amplification fragments were evaluated by agarose gel electrophoresis (1.8% w/v TE buffer). Bands indicated in **Fig 2B** were excised with a scalpel, purified using Zymoclean Gel DNA Recovery Kit (Zymo Research) and cloned into TOPO TA Cloning vectors (Thermo Fisher Scientific).

### Confocal imaging of E49>GFP, shRNA thoracic ganglia

F1 males flies from E49>GFP, UAS-shRNA crosses were anesthetized, then briefly dipped in 95% EtOH and pinned to a dissecting plate. The thoracic ganglia from at least 3 flies was removed and immediately placed in cold 4% PFA/1x PBST (phosphate buffered saline + 2% Triton X-100 + 10% Normal Goat Serum) for 15 minutes, then degassed in fixative at room temperature for 45 minutes. 3 washes with PBST (10 minutes each) were performed, and ganglia were incubated with a mouse anti-GFP 1° antibody (1:250 dilution MilliporeSigma #11814460001) in 1x PBST (0.2% Triton X-100 + 10% Normal Goat Serum) overnight at 4°C. Ganglia were then washed 3 times (10 minutes each) with 1x PBS and then incubated with 2° antibody (1:250 dilution of Alexa Fluor 488-conjugated goat anti-mouse, Thermo Fisher Scientific #A-11029) in 1x PBST (0.2% Triton X-100 + 10% Normal Goat Serum) for 1 hour at room temperature, then washed with 1x PBST 3 times (10 minutes each) and placed on a concave dissecting dish. The PBST was removed, and the tissue was briefly rinsed with 1x PBS, then as much of the liquid was removed as possible with a pipette before adding FocusClear (CelExplorer) directly to the tissue. After tissue clearing occurred, the ganglia were transferred to a slide with a drop of ProLong Diamond AntiFade Mountant with DAPI (Thermo Fisher Scientific) and a glass coverslip placed over the preparation. The cover was glass was sealed with nail polish and allowed to set overnight in a dark 4°C refrigerator. Images were generated with a Zeiss Confocal microscope.

### Fly stocks

See **S5 Table** for all *Drosophila* stocks used in this study. TRiP stocks were not treated for the possible presence of *Wolbachia*.

## Supporting information

S1 Table Retesting of non-rescuing lines to determine false positive rate. (XLSX)

S2 Table Comprehensive screen data. Sheet 1: first screen of full TRiP line assembly. Sheet 2: retest summary of candidate rescues for validation.
(XLSX)

S3 Table Sheet 1: computational output showing gRNA properties. Sheet 2: gRNA oligonucleotide sequences ordered for cloning into pCFD3.1 vector.
(XLSX)

S4 Table PCR Primers for Cas9 cutting validation. (XLSX)

S5 Table *Drosophila* stocks used in this study. (XLSX)

S1 Fig Introgression scheme. TRiP lines carry the vermillion (v) marker on their AttP2 integration site. Initial setup: virgin w^1118^ with a miniwhite transgene inserted into AttP2 were crossed to males from the desired TRiP line. F1 offspring males heterozygous on the third chromosome were again crossed to virgins from the w^1118^ background to yield F2 virgin females homozygous on the X for w- and heterozygous on the 3^rd^ chromosome for introgression. Introgression and backcrossing: F2 virgin females heterozygous on the third (very light orange eyes) were backcrossed to males from the w1118 background. This was repeated with the every subsequent generation (F3-F6) of females heterozygous on the third to gradually replace more of the 3^rd^ chromosome containing the AttP2<shRNA> with the third chromosome from the w1118 background. Sibling cross: after 6 generations of introgression, male and female siblings with light orange eyes were crossed to each other, and F7 offspring with white eyes were selected (completely lacking miniwhite in the AttP2 site, indicating presence of shRNA).
(PDF)

S2 Fig Confocal microscopy of thoracic ganglia from E49>GFP flies expressing rescuing shRNAs. All images are single-plane on 10X objective. shRNAs were (A) *msk*, (B) *Su(Tpl)*, (C) *SF2*, (D) *Polybromo*.
(TIFF)

S3 Fig Western immunoblot of whole fly heads against hTDP-43 and tubulin (loading control). Lanes indicate whether a control shRNA (mCherry) or the shRNA against *SF2* was used. The hTDP-43 transgene and shRNA were induced using the TubGAL80^TS^ system (by switching from 21°C to 29°C) and controlled by the pan-glial *Repo-GAL4* driver. (a) Chemiluminescent output of blots on two separate experiments. Top blot used rabbit polyclonal hTDP-43 antibody (target band indicated by arrow), The bottom blot used anti alpha-tubulin as a loading control (indicated by arrow). The sample loaded on each lane is indicated at the top. 2U (wild type cantonized control strain) and non-induced (21°C) samples were included as negative controls. (b) Quantitative assessment of hTDP-43 band intensity was performed using ImageJ and normalizing to alpha-tubulin of the 2U sample. Intensity is average across the two experiments. Error bars: SEM. Statistical significance was assessed by unpaired two-tailed Student’s T-test (95% CI). Significant changes were detected in *SF2* sample after induction (indicated by *, P < 0.05). induction with the mCherry-shRNA control showed a trend that was not significantly different in the two experiments. No significant differences in hTDP-43 induction were detected when mCherry control shRNA and *SF2* shRNA induced samples were compared.
(TIF)
