# Supplementary figures and images for: A behavioral screen for mediators of age-dependent TDP-43 neurodegeneration identifies SF2/SRSF1 among a group of potent suppressors in both neurons and glia
Source: PLoS Genet. 2021 Nov 1;17(11):e1009882. doi: 10.1371/journal.pgen.1009882 (PMC8584670; doi:10.1371/journal.pgen.1009882)

Supplemental Figure 1

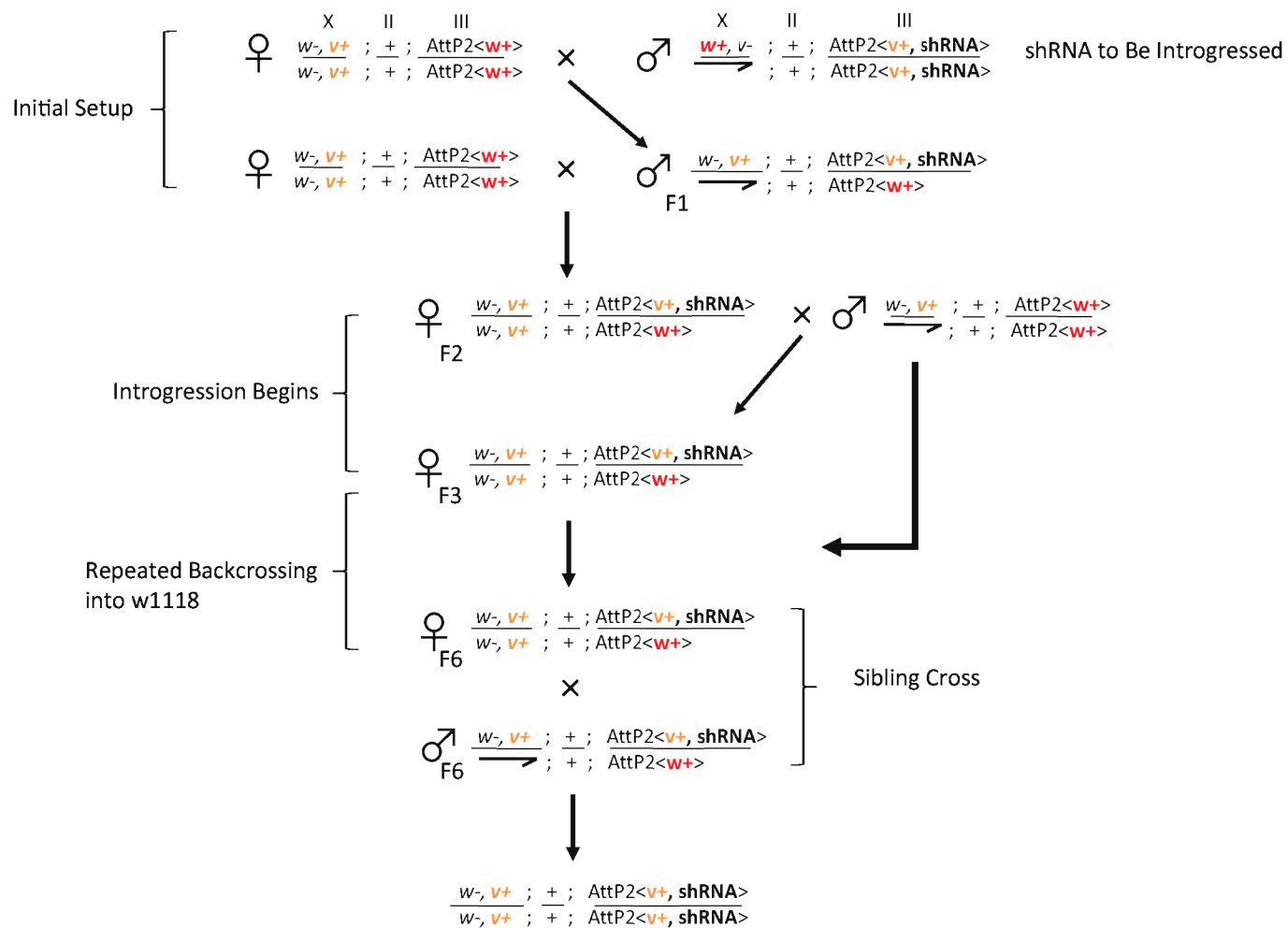

Supplement: S1 Fig — TRiP lines carry the vermillion (v) marker on their AttP2 integration site. Initial setup: virgin w1118 with a miniwhite transgene inserted into AttP2 were crossed to males from the desired TRiP line. F1 offspring males heterozygous on the third chromosome were again crossed to virgins from the w1118 background to yield F2 virgin females homozygous on the X for w- and heterozygous on the 3rd chromosome for introgression. Introgression and backcrossing: F2 virgin females heterozygous on the third (very light orange eyes) were backcrossed to males from the w1118 background. This was repeated with the every subsequent generation (F3-F6) of females heterozygous on the third to gradually replace more of the 3rd chromosome containing the AttP2 with the third chromosome from the w1118 background. Sibling cross: after 6 generations of introgression, male and female siblings with light orange eyes were crossed to each other, and F7 offspring with white eyes were selected (completely lacking miniwhite in the AttP2 site, indicating presence of shRNA). (PDF) [file pgen.1009882.s006.pdf]

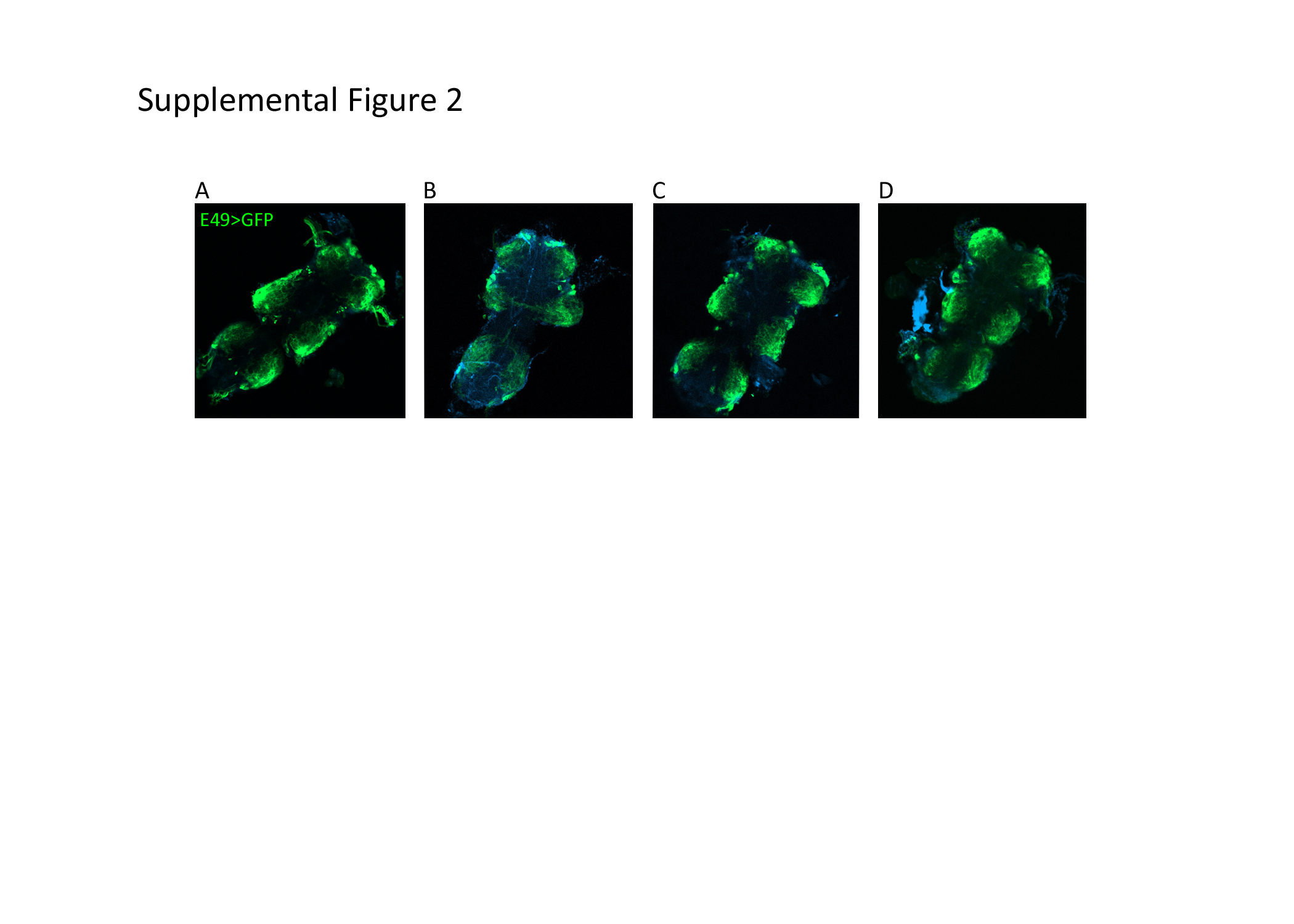

Supplement: S2 Fig — All images are single-plane on 10X objective. shRNAs were (A) msk, (B) Su(Tpl), (C) SF2, (D) Polybromo. (TIFF) [file pgen.1009882.s007.tiff]

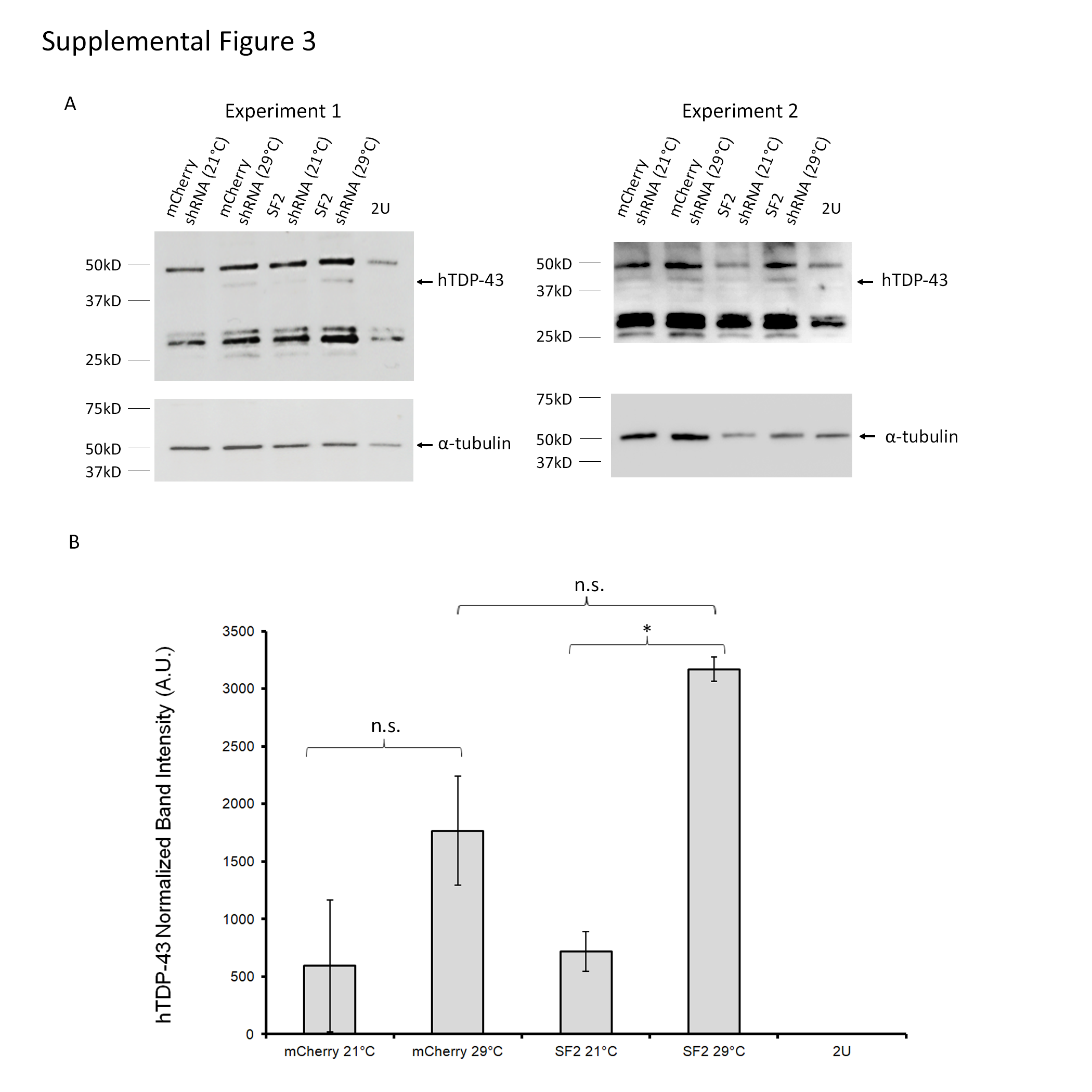

Supplement: S3 Fig — Lanes indicate whether a control shRNA (mCherry) or the shRNA against SF2 was used. The hTDP-43 transgene and shRNA were induced using the TubGAL80TS system (by switching from 21°C to 29°C) and controlled by the pan-glial Repo-GAL4 driver. (a) Chemiluminescent output of blots on two separate experiments. Top blot used rabbit polyclonal hTDP-43 antibody (target band indicated by arrow), The bottom blot used anti alpha-tubulin as a loading control (indicated by arrow). The sample loaded on each lane is indicated at the top. 2U (wild type cantonized control strain) and non-induced (21°C) samples were included as negative controls. (b) Quantitative assessment of hTDP-43 band intensity was performed using ImageJ and normalizing to alpha-tubulin of the 2U sample. Intensity is average across the two experiments. Error bars: SEM. Statistical significance was assessed by unpaired two-tailed Student’s T-test (95% CI). Significant changes were detected in SF2 sample after induction (indicated by *, P < 0.05). induction with the mCherry-shRNA control showed a trend that was not significantly different in the two experiments. No significant differences in hTDP-43 induction were detected when mCherry control shRNA and SF2 shRNA induced samples were compared. (TIF) [file pgen.1009882.s008.tif]
